# Insights About Nitazene Use From Self-reported Experiences on Reddit Obtained Using Natural Language Processing

**DOI:** 10.1097/ADM.0000000000001760

**Published:** 2026-09-18

**Authors:** Pranav Sompalle, Jeanmarie Perrone, Rachel Wightman, Jennifer Love, JaMor Hairston, Sahithi Lakamana, Abeed Sarker

**Affiliations:** Department of Chemistry, University of Pennsylvania School of Arts and Sciences, Philadelphia, PA (PS); Department of Emergency Medicine, University of Pennsylvania Perelman School of Medicine, Philadelphia, PA (JP); Center for Addiction Medicine and Policy, Penn Medicine, Philadelphia, PA (JP); Department of Emergency Medicine, Brown University Alpert Medical School, Providence, RI (RW); Department of Emergency Medicine, Mount Sinai Icahn School of Medicine, New York, NY (JL); and Department of Biomedical Informatics, Emory University School of Medicine, Atlanta, GA (JH, SL, AS).

**Keywords:** nitazene, synthetic opioids, substance-related disorders, natural language processing, social media, novel psychoactive substances

## Abstract

**Introduction:**

Nitazenes are an emerging class of highly potent synthetic opioids not approved for clinical use. Knowledge of nitazene-associated practices and clinical effects remains limited. Reddit contains nitazene-related discussions describing self-reported experiences. We analyzed nitazene mentions to characterize these experiences.

**Methods:**

Nitazene-mentioning posts between 01/2019 and 10/2025 were extracted via natural language processing-based lexical filters, and a random sample of 293 mentions was qualitatively reviewed and annotated for themes consolidated via consensus discussion. Repeated findings more specific than themes were synthesized as subthemes, and a validated GPT-4o-based pipeline was applied to all mentions to forecast the theme distribution.

**Results:**

A total of 31,638 mentions were extracted; frequency increased between 2022 and 2024 (mean 584 mentions/month, *P* < 0.01 increase compared with 2019–2021’s mean of 197 mentions/month). Ten themes were curated, with source (31.3%), potency and dose (26.5%), and uncertainty (25.9%) most prevalent in the annotated sample. Across the full dataset, automated characterization predicted that general experience (44.7%), general comment (37.9%), and potency and dose (27.6%) were the most prevalent thematic labels. Nine subthemes included information about counterfeit pills, withdrawal management difficulties, motivating factors for use, benzodiazepine adulteration, and vascular injury associated with intravenous administration.

**Discussion:**

Increasing nitazene mentions on Reddit may reflect increasing interest and use. Thematic characterization identified patterns in nitazene use, effects, and distribution relevant to clinical practice and public health. Insights provided by subthemes align with laboratory potency assessments and emerging evidence from case series and health alerts.

**Conclusion:**

These findings can inform public health monitoring, overdose prevention, and clinical characterizations of nitazene use.

Nitazenes are a class of nonfentanyl novel synthetic opioids (NSOs) that act as potent agonists of the mu opioid receptor (MOR).^1–3^ Pharmaceutical research into the development of nitazenes, first synthesized in the 1950s, was halted because of their extreme potency and tentative addiction risk.^4^ Nitazenes have been shown to bind MORs with greater affinity than fentanyl and exhibit potencies up to 800x that of morphine and 40x that of fentanyl, with equipotency relative to fentanyl observed at minimum.^2,5^ Nitazene variants identified in nonfatal overdoses and decedent toxicology reports include isotonitazene, metonitazene, protonitazene, N-pyrrolidino etonitazene (etonitazepyne), and N-desethyl isotonitazene.^2,5,6^ Having never been approved for clinical use, nitazenes first emerged in the U.S. in 2019 and have been detected predominantly in the Midwest, South, and Northeast, including Philadelphia since 2022.^6–8^ Moreover, nitazenes have been identified in over 250 drug overdose deaths since 2019, contributing to the growing detection of nonfentanyl NSOs in fatal and nonfatal overdoses.^9^ However, nitazenes are not detectable on standard drug screens, which can make routine identification difficult.^4^ These developments have resulted in the scheduling of ten nitazene variants, but numerous novel nitazene analogs remain unregulated, including N-desethyl isotonitazene.^9^

Despite the recent detection of nitazenes in human exposures, behaviors and clinical implications associated with nitazene use are not well characterized. The population that uses nitazenes remains unclear, with characterizations limited to inferences that individuals seeking other opioids may unintentionally use nitazenes; these inferences are informed by recent drug checking services and seizures increasingly detecting nitazenes in illicit heroin/fentanyl.^5^ As MOR agonists, nitazene-involved overdoses are effectively reversed with naloxone, but a minimum effective naloxone dosage and naloxone dosage differences between nitazene-involved and fentanyl-involved overdoses remain unclear.^3,6,10^ Anecdotal experiences have suggested that symptoms associated with withdrawal from nitazenes arise faster and are more severe than those associated with withdrawal from fentanyl; additionally, metonitazene-involved ODs have been suggested to heighten risk for concurrent cardiac arrest.^5,6^ These limited data on nitazene-associated behaviors and clinical effects are concerning in the context of increasing detection of nitazenes in the illicit drug supply and in overdose deaths, highlighting the need for expanded study into nitazene use.^8,9,11^

Self-reported experiences on moderated, anonymous online discussion forums such as Reddit have emerged as complementary tools for drug surveillance and for understanding experiences with emerging substances. The National Drug Early Warning System (NDEWS) operates an online monitoring platform that tracks drug-related keywords across ~80 drug-oriented communities on Reddit,^11^ and insights obtained from Reddit have been previously reported to predict toxicology trends.^12^ In the context of nitazenes, for example, NDEWS has identified increasing nitazene mentions on Reddit between 2023 and 2024 and discussions about nitazene-associated withdrawal, tolerance, and exposure.^11^ Other studies have likewise leveraged online communities such as Reddit to characterize emerging cannabis use patterns,^13,14^ opioid treatment and withdrawal discussions,^15,16^ increasing fentanyl-related discussion across substance use forums as a marker of changes in the drug supply,^17^ and self-reported experiences and behavior change related to xylazine exposure.^17^ Moreover, application of natural language processing (NLP) to Reddit comments and posts has enabled extraction of large quantities of data containing information about specific drugs such as xylazine, buprenorphine, and fentanyl.^15,18^ Analysis of self-reported experiences from Reddit and the application of NLP to extract data may offer timely insights into nitazene use.

In this study, we apply NLP to Reddit conversations to identify discussions containing nitazene-related keywords and quantify interest in nitazene variants over time. We then integrate clinical toxicology and addiction medicine expertise to develop a standardized thematic framework through which these NLP-extracted self-reported experiences are characterized to explore poster-reported practices and clinical effects associated with nitazene use.

## METHODS

### NLP-based Extraction of Nitazene-related Reddit Mentions and Frequency Analysis

This study was reviewed by the Emory University Institutional Review Board and considered exempt (category 4; publicly available data). We collected Reddit data dated 01/2019–04/2024 using the pushshift.io API but collected data dated 04/2024–10/2025 using the Python Reddit API Wrapper (PRAW) because Reddit API changes altered pushshift.io availability. Using 35 nitazene-related keywords capturing lexical variants of nitazene names obtained using NLP (word2vec and DeepSeek) and filtered by author RW (toxicology expertise) (Table 1),^19^ we collected posts containing at least one keyword and associated comments available at the time of extraction between January 2019 and October 2025 (Supplemental Section S1, https://links.lww.com/JAM/A851). Mention frequencies were grouped by month (Fig. 1), and average monthly mention rates between 2019–2021 and 2022–2024 were compared using the Mann-Whitney *U* test.

### Qualitative Review of Nitazene-related Reddit Mentions and Thematic Analysis of Self-reported Experiences

A pilot sample of 60 mentions was reviewed by authors RW and JP for quality control and preliminary framework development. An independent random sample of 293 mentions was then annotated by authors JH and PS, and preliminary categories were iteratively refined through consensus discussion involving PS, RW, and JP to generate finalized themes (Table 2). Authors JH and PS then re-characterized the 293 mentions using these finalized themes, with multiple themes permitted per mention; JP and PS reviewed theme assignments for consistency and conducted a secondary review of theme-assigned mentions to identify novel information (grouped as “subthemes”) pertinent to each category and more specific in scope than the parent themes (Table 3). Additional detail is provided in Supplemental Section S2, https://links.lww.com/JAM/A851.

To determine whether the thematic distribution observed in the manually annotated sample extended to the full dataset, we applied the validated GPT-4o-based annotation pipeline developed by Hairston et al^20^ to all 31,638 extracted mentions (Fig. 2). Because 2 manually annotated posts were used during few-shot prompting, these posts were excluded from evaluation of automated performance. On the remaining 291 held-out manually annotated mentions, we evaluated label-specific accuracy, precision, recall, F1, specificity, and balanced accuracy; detailed performance results are provided in Supplemental Section S5, https://links.lww.com/JAM/A851 and Supplemental Table S2, https://links.lww.com/JAM/A851. The pipeline then generated binary predictions across the finalized thematic labels for the full dataset, and predicted positive counts and rates were aggregated for each label. Detailed methods about this automated characterization are provided in Supplemental Section S3, https://links.lww.com/JAM/A851.

## RESULTS

### NLP-based Extraction of Nitazene Mentions on Reddit Yields Extensive Dataset of Self-reported Experiences That Suggest Increasing Use or Interest

We extracted 31,638 mentions containing at least one nitazene keyword, comprising 5519 posts and 26,119 comments under these posts, publicly available at the time of collection (Supplemental Section S1, https://links.lww.com/JAM/A851). Among the 27,547 mentions with non-deleted authors, comprising 5129 posts and 22,418 comments, there were 7897 unique Reddit users, including 2175 unique posters and 6,435 unique commenters. Across all 5519 extracted posts, mean post length was 751.63 characters (standard deviation = 2297.63), while mean comment length across all 26,119 extracted comments was 292.48 characters (standard deviation = 477.10). These posts and comments appeared across 521 distinct named subreddits, with mentions concentrated in opioid- and research chemical-focused communities such as “Opioid_RCs,” “researchchemicals,” “opiates,” “heroin,” “fentanyl,” and “OpiatesRecovery” (Supplemental Table S1, https://links.lww.com/JAM/A851).

We observed increases in monthly frequencies of posts and comments under posts starting in February 2022 and May 2023, respectively (Fig. 1). Combined, an average of 584 nitazene mentions were posted per month between January 2022 and December 2024, relative to 2019–2021’s average of 197 mentions/month (*P* = 5.89e-7 difference between 2019–2021 and 2022–2024, U = 1092, Mann-Whitney *U* test).

### Thematic Analysis of Nitazene-related Reddit Mentions Identifies 10 Themes and 9 Subthemes That Reveal Insights into Nitazene Use Practices and Clinical Effects

Iterative refinement of preliminary thematic categories yielded ten consensus themes, which we used to characterize mentions in the *N* = 293 sample (Table 2). Systematic review of each theme’s set of mentions from the sample revealed 9 subthemes that emerged from multiple mentions and described novel knowledge related to nitazenes (Table 3).

### NLP-based Forecasting of Thematic Labels Across the Full 31,638-mention Dataset

On the held-out 291-mention evaluation set, average precision, recall, F1, specificity, and balanced accuracy across labels were 0.58, 0.65, 0.57, 0.87, and 0.76, respectively, although performance varied across themes (Supplemental Table S2, https://links.lww.com/JAM/A851, Supplemental Fig. S1, https://links.lww.com/JAM/A851). We therefore report full-dataset automated outputs as predicted label frequencies rather than prevalence estimates. Across the full dataset, predicted positive rates were highest for general experience (44.7%), general comment (37.9%), and potency and dose (27.6%). Detailed label-specific automated frequencies, held-out evaluation metrics, and bootstrap-based uncertainty analyses are provided in Supplemental Sections S5–6, https://links.lww.com/JAM/A851.

### Self-reported Sourcing and Patterns of use

Posters reported sources through which they obtain nitazenes in 92 mentions (31.3%); many described illegal online vendors selling prescription opioid pills containing nitazenes mixed with other substances (ie, “pressed pills”) (*n* = 7 mentions). One poster, for example, described purchasing pills that were “according to the vendor 20 mg Oxymorphone pills containing Metonitazene.” Posters also identified nitazenes as adulterants of illicit benzodiazepines, including diazepam, bromazolam, etizolam, and alprazolam (*n* = 4). One poster additionally described a multidrug overdose that they attributed to isotonitazene mixed with a benzodiazepine: “And it wasn’t a single agent overdose … still ISO [isotonitazene] mixed with benzos is the best explanation I have.” Additional vendor-labeled and benzodiazepine-adulteration quotations are provided in Supplemental Section S4.1, https://links.lww.com/JAM/A851.

Mentions of international sources, regional variation in nitazene access, or differences between various countries’ regulations on nitazenes were present in 30 (10.2%). These mentions most often described geographically specific online sourcing, including Chinese suppliers, European vendors, dark-web purchasing, and region-specific availability of nitazene variants (Supplemental Section S4.2, https://links.lww.com/JAM/A851).

### Self-reported Potency, Administration, Withdrawal, and Management Challenges

A total of 78 mentions (26.5%) documented poster-reported assessments of the potency of nitazene variants and experiences with particular dosages for different nitazene variants. One poster, for example, specifically described potencies of nitazene variants alone and when mixed with other substances (eg, 1:2 etodesnitazene:caffeine) relative to morphine:

“…a ratio of 1 part of Etodesnitazene (aka “EtaZene”) to 2 parts of Caffeine, exhibiting a potency of ~25x that of Morphine, means that pure Etazene powder has got a potency of around 75x Morphine: (75 / (1 + 2)) = 25…”

A total of 76 (25.9%) mentions described uncertainty concerning the consequences and management of nitazene use. Reported uncertainty included questions about withdrawal symptoms, withdrawal treatment, whether to dispose of nitazenes, and dosage recommendations. For example, one poster probed the consequences of taking protonitazene in combination with buprenorphine: “…Out of opioids … got couple 80 mg oxycontin … to hold off [withdrawal] and … 3 dirty 30s with [protonitazene] in. Can I take [buprenorphine] with [protonitazene] ?…” Additional uncertainty-related quotations are provided in Supplemental Section S4.3, https://links.lww.com/JAM/A851.

A total of 54 mentions (18.4%) described withdrawal symptoms or side effects associated with nitazene use, including those specific to particular variants. Reported withdrawal symptoms common across metonitazepyne, metonitazene, and protonitazene use aligned with withdrawal symptoms from other opioids and included fever-like symptoms (shivering, increased body temperature), pain around the teeth, worsened digestion, cramps, spasms, vomiting, and insomnia. Some posters also identified nitazene withdrawal as having an early onset nature (within a few hours after last nitazene administration) (*n* = 3) that may distinguish it from withdrawal associated with other opioids, including fentanyl. Additional withdrawal-related detail is provided in Supplemental Section S4.4, https://links.lww.com/JAM/A851.

Posters described difficulties managing nitazene withdrawal with both medications for opioid use disorder and less potent illicit opioids (*n* = 4), including buprenorphine inefficacy in metonitazene withdrawal and reduced efficacy of fentanyl and heroin for withdrawal associated with other nitazene variants. Some posters also reported increased tolerance to lower-potency opioids after using nitazenes (*n* = 2; Supplemental Section S4.4, https://links.lww.com/JAM/A851).

Posters also described nitazene preparation practices and routes of administration in 52 (17.7%) mentions. Routes of administration included oral and intranasal administration for metonitazene and intravenous, intranasal, or inhaled administration for other variants, including etonitazene and metonitazepyne. Poster-reported preparation practices were limited to mixing nitazenes with other substances, and some posters described route of administration-dependent potency or preparations specific to intravenous use: “…If the product is in the form of a salt or a salt mix with caffeine or mannitol, it will work [intravenously].”

A total of 38 (12.9%) mentions described polysubstance use involving nitazenes. Reported combinations of substances used simultaneously or over the same time period included metonitazene, etonitazepyne, and cocaine; metonitazene and amphetamine; isotonitazepyne, N-desethyl isotonitazene, protonitazepyne, and ketamine; metonitazene and lidocaine as an adulterant; and various nitazene variants with O-desmethyltramadol (O-DSMT). Several posters specifically described simultaneous nitazene and O-DSMT use to heighten euphoria, and some posters asked whether substances such as kratom could be used to supplement methadone or buprenorphine during withdrawal.

Nitazene variant comparisons appeared in 31 (10.5%) mentions and reflected posters’ experiences or understandings of different variants’ relative effects and potencies. In addition, 33 (11.2%) mentions described general experiences with nitazenes without detailed discussion of withdrawal, side effects, or potency. Some mentions identified a short duration of effects or euphoria as a feature distinguishing nitazenes from other opioids (*n* = 2). General comments appeared in 69 (23.5%) mentions and often consisted of contextual or knowledge-sharing material, including links to other discussions or external resources, brief variant references, or commentary on treatment access, policy, overdose prevention, or vendor practices. Additional detail on general comment and general experience content is provided in Supplemental Section S4.2, https://links.lww.com/JAM/A851.

### Motivations for Use, Tolerance, and Complications

Several posters described nitazenes’ higher potency relative to fentanyl, heroin, oxycodone, and kratom, as well as increased tolerance for some conventional opioids, as motivating factors driving nitazene use (*n* = 4). One poster described metonitazepyne use to cope with increased oxycodone tolerance (see Supplemental Section S4.5, https://links.lww.com/JAM/A851). Other motivating factors identified by posters included nitazenes’ reduced cost relative to fentanyl and the ability to avoid detection on employer-administered drug tests while using nitazenes (*n* = 2 mentions).

Some posters reported vascular injury and venous irritation (“vein burning”) associated with intravenous administration of nitazenes (*n* = 4), with etodesnitazene (etazene) and metonitazene described as producing greater burning sensations. Posters also described attempts to lessen burns by modifying preparations with baking soda: “…realistically all of the zenes are not very [intravenously] safe … needed a baking soda buffer to lessen the burn …” Additional quotations are provided in Supplemental Section S4.5, https://links.lww.com/JAM/A851.

## DISCUSSION

### Principal Findings

We characterized nitazene-related Reddit experiences into 10 consensus themes and 9 subthemes describing clinical effects, usage practices, and distribution patterns. Descriptions of sources and vendors, potency and dose, and uncertainty were among the most common themes in the manually annotated sample, while application of the NLP-based annotation pipeline to all 31,638 mentions further suggested that general experiences, general comments, and potency and dose remained the most prevalent thematic labels across the broader dataset.

In particular, mentions assessing potency and dosages of distinct nitazene variants are consistent with prior evidence of high MOR activity and may also point to meaningful differences between variants, including user-described attempts to quantify or modulate potency through mixtures such as etodesnitazene-caffeine formulations.^1,3,7^

Uncertainty concerning nitazenes and their potency, dosing, withdrawal management, and safety for concurrent use with medications like buprenorphine was coded in 76 mentions (25.9% of the 293-mention sample). This pattern suggests that public health, health literacy, and overdose prevention programs may need to expand their services to provide timely, nitazene-specific information on overdose risk, variable potency, dosing uncertainty, withdrawal recognition, and questions surrounding self-management or concurrent use with medications such as buprenorphine. More broadly, these findings suggest that people who use drugs may actively seek practical guidance about nitazenes in settings where formal clinical or public health information remains limited. This uncertainty is characteristic of an evolving illicit synthetic opioid supply, in which posters rely on others’ self-reported experiences documented in social media to inform personal overdose prevention and safe practices.^12,18^

Posters also reported motivating factors driving nitazene use: higher potency compared with fentanyl and heroin, lower cost relative to fentanyl, and detection avoidance on routine drug testing. Importantly, these reports suggest that nitazene use cannot be assumed to be uniformly inadvertent and that some posters may be actively seeking nitazenes for these factors, which has possible implications for overdose prevention and treatment efforts that may need to address intentional nitazene use in addition to unintentional exposure. Poster-reported use of intravenous, intranasal, or inhaled routes of administration depending on nitazene variant align with previous studies’ identification of nitazene administration via vapes, pills, dissolved powders, and nasal sprays.^3,10^ However, novel reports of “vein burning,” comprising vascular injury and veinous irritation, associated with intravenous use of nitazenes could suggest the presence of potential adulterants in formulations that may present additional harms.

Withdrawal experiences shared by posters mirror established patterns of opioid dependence with symptoms of gastrointestinal distress and insomnia, and mentions of an earlier symptom onset profile (few hours), as reported by some posters. These results add to previously published clinical observations that highlight the similarities of nitazene withdrawal to those of conventional opioids but lack insight into its temporal profile.^3,12^ This insight into an early onset profile, however, is consistent with preliminary, community-based observations presented in a Philadelphia-based health alert.^5^ The self-reported lack of efficacy of buprenorphine to relieve withdrawal symptoms in some cases may reflect a consequence of a highly heterogeneous illicit drug supply or challenges observed in other contexts such as reports of standard buprenorphine doses being inadequate in controlling withdrawal symptoms among patients with high levels of tolerance associated with fentanyl.^21^ Self-reported experiences with increased tolerance to less potent opioids like heroin and fentanyl following nitazene use further substantiate challenges with nitazene withdrawal management. Taken together, these reports suggest that clinicians and overdose prevention programs may need to anticipate the potential for rapid withdrawal onset and high opioid tolerance in nitazene use cases. Although these observations do not identify optimal treatment strategies, they do suggest that nitazene exposure could be considered when withdrawal appears unusually severe or difficult to manage. These possible clinical implications require further study.

Poster reports of counterfeit prescription pills containing nitazenes (“pressed pills”) reflect the integration of nitazenes into illicit opioid markets via online vendors. The apparent ability of some posters to identify specific nitazene variants may reflect product descriptions provided by illicit online vendors and may suggest that some Reddit posters engage with illicit nitazene markets differently from people obtaining opioids through the street supply. In addition, accounts of “pressed pills” echo recent health alerts from Philadelphia documenting nitazenes in counterfeit pills.^5,9^ Repeated reports of nitazenes as adulterants of illicitly obtained benzodiazepines, including bromazolam and etizolam, are also consistent with these Philadelphia-based health alerts and raise concern for complex overdose presentations, as described by some posters.^5,9^ Clinical case series have similarly reported acute toxicity resulting from the co-ingestion of nitazenes and sedatives.^10^ Posters’ comments about simultaneous use of nitazenes and *O*-DSMT reveal interest in nitazene-involved polysubstance use.

Leveraging self-reported experiences involving nitazene use obtained from Reddit, this study reiterates the potential for monitoring of bulk Reddit data extracted using NLP as a real-time complement to toxicological surveillance systems, with prior studies also leveraging NLP to monitor novel psychoactive substances.^12,15,18^

### Limitations

Reddit demographics that may be skewed by age and ethnicity limit these findings.^22^ Changes in Reddit’s API and policies in April 2024 necessitated a transition in data collection source (from the pushshift.io API to PRAW) and created variations in the number of mentions that could be extracted, potentially limiting conclusions from monthly mention frequencies, in particular. Because posters can remove their data at any time, and the use of several rounds of data collection followed by analysis at a later time, extracted mentions are subject to variable public accessibility. Another limitation is that the automated pipeline could over- or under-represent specific themes, especially lower-performing labels, and full-dataset characterization should therefore be interpreted descriptively. A further limitation is that it is not possible to verify the validity of self-reported experiences obtained from Reddit. In addition, we synthesized subthemes via secondary review of theme-assigned mentions involving a subset of the reviewers, meaning that possible inter-reader variability may have affected how repeated findings were characterized. We also could not determine geographic location for most posters, which limited regional interpretation of the findings. Furthermore, we designed our nitazene keyword set to emphasize variant names and lexical variants rather than sometimes ambiguous street terminology, which may have reduced capture of some nitazene-related discussions. Because qualitative characterization was performed on a manually reviewed sub-sample, these findings should be interpreted as preliminary. Finally, specific characteristics associated with nitazene use derived from self-reported experiences may not be uniformly reflected across all nitazene posters. However, the convergence of multiple experiences’ characteristics describing consistent phenomena may offer validity to these observations. Integration of these emerging preliminary results with laboratory and clinical toxicology reports^1–3,10^ can refine our understanding of nitazene pharmacodynamics, inform evidence-based clinical protocols, and aid efforts to track the emergence of nitazenes into illicit opioid distribution across the United States.

## CONCLUSION

This study leverages NLP to extract Reddit mentions describing self-reported experiences with nitazenes and uses coding and thematic characterization to interpret these experiences and identify emerging trends related to nitazene usage practices, clinical effects, and distribution. Large proportions of mentions describe illicit sources, potency and dosages, and uncertainty concerning nitazenes. We also reveal preliminary insights relevant to clinical management, public health interventions, and overdose prevention related to nitazene use and distribution, including “pressed pills” as a common mode of distribution, “vein burning” comprising vascular injury and venous irritation associated with intravenous administration, adulteration patterns involving benzodiazepines, variable efficacy of medications for opioid use disorder treatment and less potent opioids in managing nitazene withdrawal, early onset of withdrawal as a potential clinical marker of nitazene use, and high potency and lower cost as motivating factors for use among posters with increased tolerance to conventional opioids.

## Supplementary Material

supplement

Supplemental Digital Content is available for this article. Direct URL citations are provided in the HTML and PDF versions of this article on the journal’s website, www.journaladdictionmedicine.com.

## Figures and Tables

**FIGURE 1.**
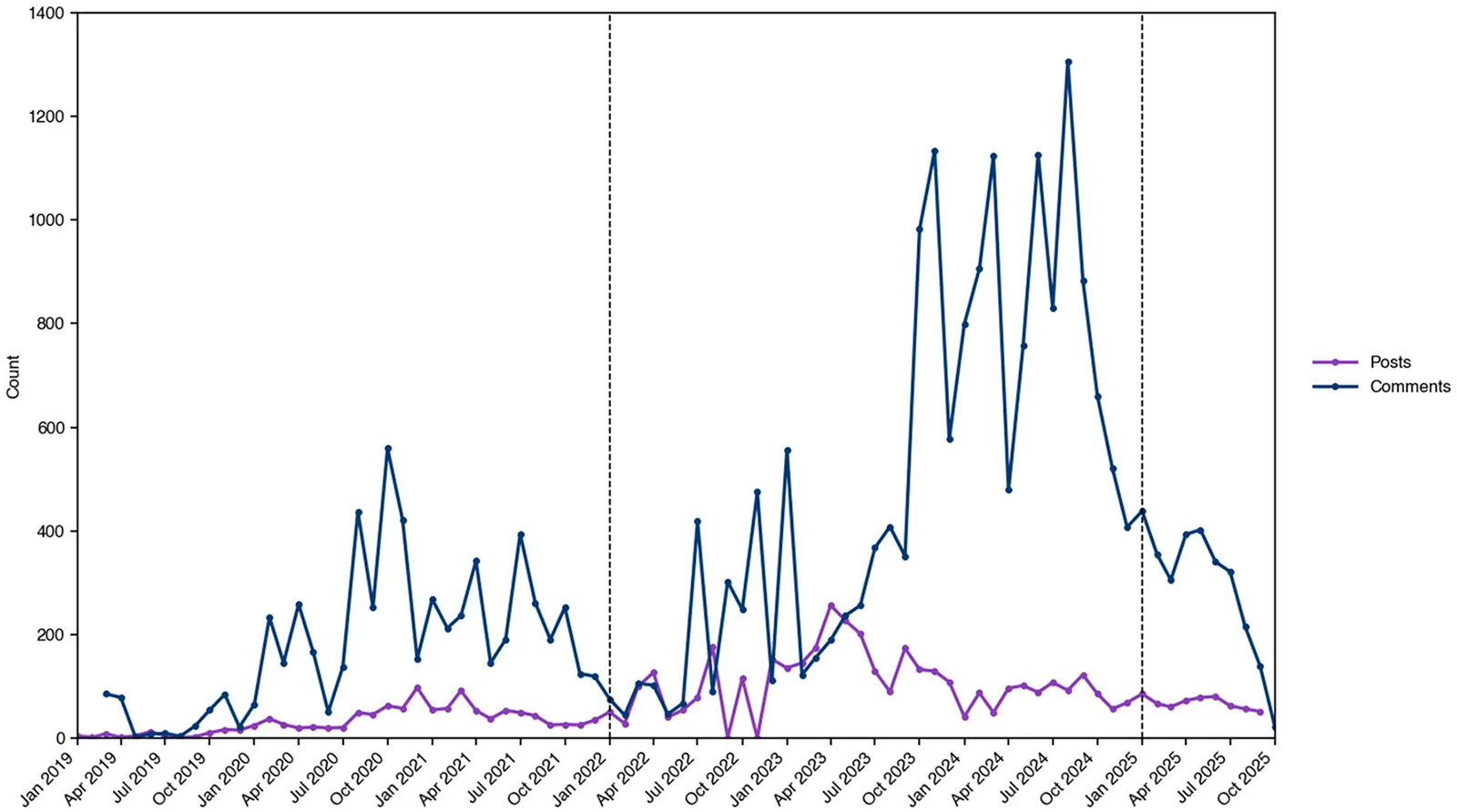
Month-wise counts of posts (5519 total, purple line) and comments (26119 total, dark blue line) extracted from Reddit using NLP fitted with keywords associated with different nitazene variants (Table 1). The time window used to extract these mentions was January 2019 to October 2025. Dashed lines demarcate January 2022 and January 2025 to visualize the 2022–2024 (all months included) time window during which 66.5% of all mentions collected in this dataset were posted.

**FIGURE 2.**
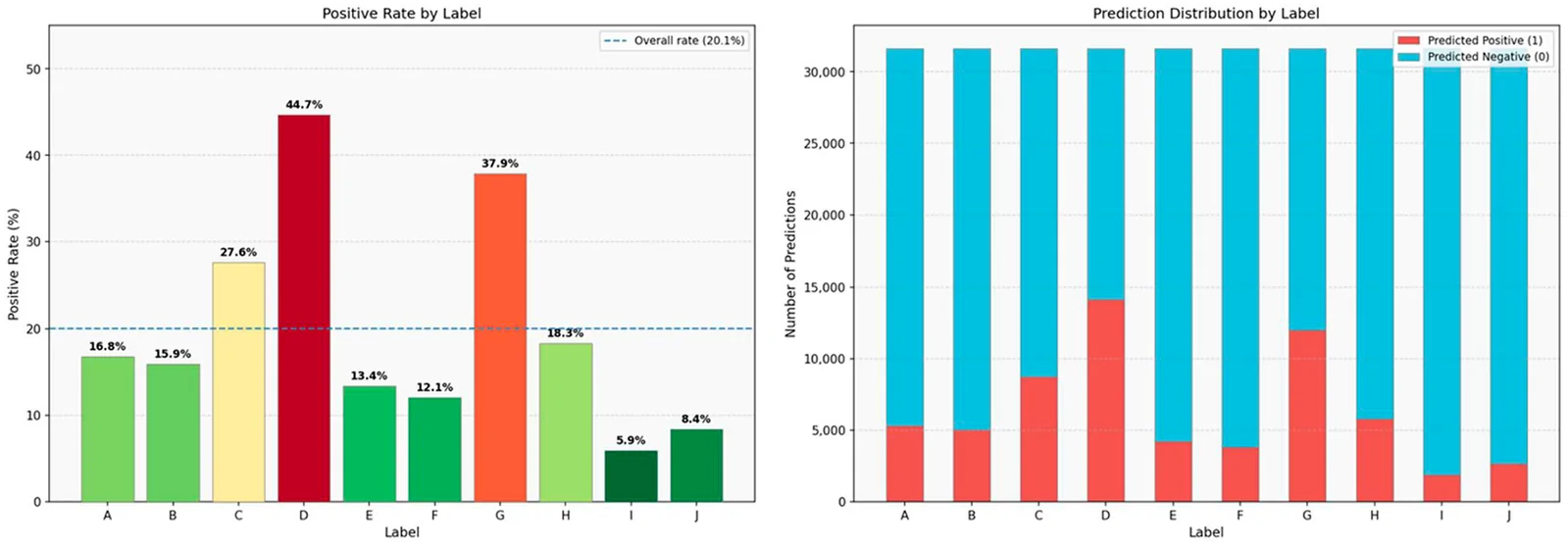
Distribution of themes predicted across the entire 31,638-mention dataset by an NLP (GPT-4o)-based annotation approach. Left, percentage of mentions predicted positive for each label, with the dashed line denoting the overall positive rate across all label-level predictions (20.1%). Right, counts of predicted positive and negative mentions by label. Labels refer to the following thematic categories: (A) Preparation practices and route of administration; (B) Source; (C) Potency and dose; (D) General experience; (E) Withdrawal and side effects experience; (F) Differences within nitazene class; (G) General comment; (H) Uncertainty about nitazenes; (I) Use of other substances alongside nitazenes; (J) International.

**TABLE 1.** Lexical Variants (Keywords) of Distinct Nitazene Variants used to Fit Natural Language Processing-based Model for Extraction of Reddit Mentions Related to Nitazene Use and Distribution

Nitazene Compound Class/Variant	Keywords Used	Count
Nitazene	*nitazene, nitazane, nitazenes, nitazines*	4
Nitazepyne	*pyne-nitazene*	1
Isotonitazene	*isotonitazene, istonitazene, isotonitazane, isonitazene, lisotonitazene*	5
Etonitazene	*etonitazene, etanitazene, etoetonitazene, etonatazine, etonitazine*	5
Metonitazene	*metonitazene, metoitonazine, metonitazine*	3
Butonitazene (butoxynitazene)	*butonitazene, butoxynitazene*	2
Protonitazene	*protonitazene, protazene, protonitazine*	3
Fluonitazene	*fluonitazene, flunitazene*	2
Clonitazene	*clonitazene*	1
Etodesnitazene (etazene)	*etodesnitazene, etazene, etezene, etizene, etozene*	5
Etomethazene	*etomethazene, etometazene, etomethazine*	3
Metodesnitazene (metazene)	*medesnitazene*	1
**Total**	**12 classes/variants**	**35 keywords**

**TABLE 2.** Frequency, Descriptions, and Examples (Quoted From Coded Mentions) of Ten Consensus Themes Identified through Iterative Refinement of Preliminary Categories during Coding of the *N* = 293 Mention Sample

Theme	Frequency of Mentions (Percentage Out of 294 Total Mentions)	Description	Example
Source	92 (31.3)	Includes reported sources of nitazenes, including vendor or country/region of availability, reported cost, and reported purity or composition.	“m/30s these days are pressed with an opiate rc such as protonitazene (most common in m30s) or another nitazene-based opioid, or even fentanyl.”
Potency and dose	78 (26.5)	Includes drug comparisons against other drugs and opioids - mainly morphine, drug strength, dosage levels	“How was your tolerance/withdrawal after blowing through the metonitazene so quickly? I started with 0.5 mg on Monday and I’m already taking 2.5 mg doses, this seems like a disaster in the making.”
Uncertainty about nitazenes	76 (25.9)	Includes questions or implied uncertainty about nitazene properties and use, such as expected potency, dosing, withdrawal management, safety, and differences between nitazene variants.	“does anybody have knowledge or experience of how physical dependency to benzimidazole opioids compares to that of conventional opioids? i have been using several different benzimidazole opioids including isotonitazene and etonitazene daily for approximately 9 – 10 months now and i’ve noticed that conventional opioids like heroin and fentanyl have little to no effect on my withdrawal symptoms anymore, no matter how high i dose them.”
General comment	69 (23.5)	Includes contextual or knowledge-sharing content peripheral to direct nitazene experiences, such as links to outside resources or brief remarks without a specific experiential topic.	“I will have to do some research, i dont know what that is”
Withdrawal and side effects experience	54 (18.4)	Includes reported withdrawal symptoms, side effects, and comments describing their onset, severity, or course.	“I was in fear of that I was going to vomit in my sleep if I was able to get any food down or swallow my tongue by accident. I remember not being able to sleep for days but I would have micro-minute naps between the cramps, spasms, dry heaving or vomiting and would wake up choking on my tongue!”
Preparation practices and route of administration	52 (17.7)	Includes route of administration, prep practices, efficacy differences	“Maybe even mix it into a pg or vg solution (ecig juice base) then vaporize that adding drops or half ML to foil or glass bowl whichever. I did this with Etazene.”
Use of other substances alongside nitazenes	38 (12.9)	Includes demonstrations of history of or interest in use of other substances alongside nitazenes during a given time period	“I have etiz, some Xanax too which I prefer to only use at night or later in the evening when I know I’m going to stay in. What I actually did was mix a little bit of metonitazine with some ODSMT powder. I enjoy the mix and it’s mostly ODSMT as I find ODSMT to be more euphoric.”
General experience	33 (11.2)	Includes general descriptions of nitazene use or interest that do not provide enough detail to be assigned to a more specific topic.	“where is […] getting any of these? 11 year NS patient here. All I got is kratom. Doesn’t work. Ugh”
Differences within nitazene class	31 (10.5)	Includes explanations or comments about the differences between nitazene variants	“Proto has longer legs and meto is pretty widely positively received for its effects, and overall characteristics.”
International	30 (10.2)	Includes descriptions of availability, usage patterns, sources, and/or markets outside of the United States	“i’m from uk and fetty dope or fetty oxy is pretty much non existant here. but there is batches of pressed zene pills going around, its like fent in strength just more euphoric i believe. (idk i never had fetty) i haven’t tried to smoke it yet but last time i tried (was diff press) it didnt run. it’s from same person and i believe its protonitazene or metonitazene.”

**TABLE 3.** Distribution and Description of Nine Specific Subthemes Synthesizing Repeated Novel Findings More Specific in Scope than themes About Nitazene Usage Practices, Distribution Patterns, and Clinical Effects

Subtheme	Theme(s) Associated With Subtheme	Frequency of Mentions
Illicitly obtained prescription opioid pills containing nitazenes mixed with other substances (“pressed pills”) are a common mode for nitazene distribution	Source; use of other substances alongside nitazenes	7
Motivating factors for using nitazenes instead of conventional opioids	General experience	6
Higher potency desired because of increased tolerance		4
Lower cost		1
Avoidance of detection on employer-administered drug tests		1
Variable efficacy of medication for opioid use disorder and less potent opioids for nitazene withdrawal management	Withdrawal and side effects experience	4
Buprenorphine inefficacy for withdrawal management		2
Inefficacy of fentanyl and heroin for nitazene withdrawal management		2
Adulteration of illicitly obtained benzodiazpeines (diazepam, bromazolam, etiazolam, and alprazolam) with nitazenes	Use of other substances alongside nitazenes	4
Veinous irritation and vascular injury (“vein burning”) associated with intravenous administration of nitazenes	Withdrawal and side effects experience	4
Early onset of nitazene withdrawal (within few hours) compared with withdrawal from conventional opioids	Withdrawal and side effects experience	3
Simultaneous use of *O*-DSMT with nitazenes to heighten euphoria	Use of other substances alongside nitazenes	3
Increased tolerance to less potent opioids following nitazene use	Withdrawal and side effects experience	2
Short duration of expected effects (eg, euphoria) is associated with nitazene use	General experience	2

Subthemes were derived from review of codes for each theme’s set of mentions from the *N* = 293 sample.
