# Supplementary material for: Insights About Nitazene Use From Self-reported Experiences on Reddit Obtained Using Natural Language Processing
Source: J Addict Med. Author manuscript; Available in PMC 2026 Nov 4. (PMC13733439; doi:10.1097/ADM.0000000000001760)
Supplement: Supplementary file 1 [file NIHMS2218347-supplement-supplement.docx]

**Supplemental Information**

**Supplemental Section S1**. Detailed overview of data extraction from Reddit, including time-dependent variability in acquisition approach.

Mentions were extracted from Reddit using NLP in three rounds: September 2023, April 2025, and October 2025. In September 2023, all mentions containing a nitazene-associated keyword (Table 1) from January 2019 onwards were extracted. In April 2025, the same framework was applied to Reddit data dated between September 2023 and April 2025. This framework was similarly used in October 2025 to extract all mentions between January 2019 and October 2025. Only mentions publicly available at the time of collection were extracted during each round, meaning that the final dataset of 31,638 mentions consists of both mentions publicly available as of October 2025 and mentions that were unavailable as of October 2025 but were publicly available between January 2019 and September 2023 or September 2023 and April 2025. All sampling used to obtain mentions for coding, iterative refinement of preliminary thematic categories, and thematic characterization was completed on the dataset formed from data extraction rounds conducted in September 2023 and April 2025. The 35 keywords were selected through an iterative, combined discovery process that incorporated internet and exploratory searches, review of selected mentions obtained by initially searching for nitazene that described additional nitazene variants and spelling/name variations, expressions semantically related to ‘nitazene’ as predicted by an NLP-based word2vec model, and additional variants proposed by DeepSeek. Ambiguous candidate terms that could refer to nitazenes were excluded during keyword curation, NLP-predicted keywords unassociated with nitazenes were filtered out by author RW, and provenance associated with each final keyword was not tracked because candidate variants were frequently identified through more than one strategy.

**Supplemental Section S2**. Additional detail on iterative sampling, thematic refinement, and subtheme generation.

A 60-mention pilot sample was first reviewed by authors RW and JP for quality control and preliminary framework development. This pilot sample size was chosen to provide a manageable set large enough to capture the range of extracted mention types and common ambiguities present in the dataset. Preliminary codes were generated inductively from this pilot review, while their organization was guided deductively by the study objective of characterizing nitazene use practices, distribution patterns, and clinical effects. An independent random sample of 293 previously unsampled mentions was then annotated by authors JH and PS. During annotation, preliminary categories were iteratively merged, split, renamed, and defined through consensus discussion involving authors PS, RW, and JP to generate the finalized framework of 10 themes. Mentions were permitted to reflect multiple themes when appropriate. Authors JP and PS then reviewed theme assignments for internal consistency, and secondary review of theme-assigned mentions yielded 9 subthemes more specific in scope than their parent themes.

**Supplemental Section S3**. NLP-based forecasting of thematic labels across the entire 31,638-mention dataset.

After validating the relevance of the themes generated from manual characterization, author JH combined the temporally separate raw data sources into a unified file. Following preprocessing, the complete dataset of 31,638 mentions extracted in this study was passed into the GPT-4o-based annotation pipeline using the validated method developed by Hairston et al. (2025). The prompts listed below were provided to the LLM pipeline. The automated characterization run processed all 31,638 mentions through the prompt pipeline in 10 hours and 33 minutes, producing 316,380 total binary predictions across the 10 finalized thematic labels, with 10 label predictions per mention, at a temperature of 0.2 to minimize response variability. In postprocessing, author JH aggregated the model outputs to compute predicted positive counts and rates for each thematic label shown in Figure 2.

Prompts:

A, Preparation practices and route of administration: “Does the post cover methods of use and preparation practices, including route of administration and efficacy differences across methods?”

B, Source: “Does the post cover the origin and acquisition of the drug, including source countries, vendors, drug costs, and purity?”

C, Potency and dose: “Does the post cover drug strength and dosing levels, including comparisons to other opioids (primarily morphine) and other substances.”

D, General experience: “Does the post cover posts that mention a personal opinion or experience with no specific identifiable topic or further elaboration?”

E, Withdrawal and side effects experience; “Does the post cover reported side effects and withdrawal symptoms associated with use?”

F, Differences within nitazene class: “Does the post cover distinctions between nitazene variants, including pharmacological or experiential differences.?”

G, General comment: “Does post share no relevant content, including generic comments or links to outside resources without meaningful context, or empty?”

H, Uncertainty about nitazenes: “Does the post cover uncertainty or limited knowledge about nitazene properties, such as expected potency, variant differences, or euphoria-inducible doses.?”

I, Use of other substances alongside nitazenes: “Does the post cover a history of or interest in using other substances alongside nitazenes within a given period?”

J, International: “Does the post reference international aspects of nitazene availability, use, or policy?”

**Supplemental Section S4**. Additional exemplar quotations and information to supplement the Results.

**Supplemental Section S4.1**. Additional quotations about vendor-labeled nitazene variants and benzodiazepine adulteration.

1. “i have a batch of pressed blue m30s coming but the vendor was upfront advising it has isotonitazene in it.”
2. “they [nitazenes] are already turning up in test batches of accord 10mg diazepam and galenika 10mg diazepam”
3. “2mg indian xanax that have [nitazene] in them they have posters all over warning about them.”
4. “And it wasn’t a single agent overdose it definitely included a benzo overdose as well as an opiate overdose and I’m sure that helped mask some [withdrawal] symptoms … still ISO [isotonitazene] mixed with benzos is the best explanation I have.”

**Supplemental Section S4.2**. Additional detail on international, general-experience, and general-comment mentions.

Mentions of international sources also included country-specific discussions of drug-checking, drug alerts, and scheduling or regulatory changes, including reports from Ireland and Australia and broader comparisons of how nitazene markets were evolving internationally.

General experiences comprised descriptions of experiences involving nitazenes, including usage experiences or demonstrated interest or a topic related to nitazenes that posters heard about, without necessarily specifying details concerning withdrawal symptoms, side effects, or dosage and potency.

General comments often consisted of contextual or knowledge-sharing material, such as links to other Reddit discussions and external resources spanning academic studies, videos, or educational content related to nitazenes rather than direct descriptions of nitazene use. Others involved brief mentions of specific nitazene variants or broader commentary on treatment access, policy, overdose prevention, or vendor practices without substantial firsthand experiential detail.

**Supplemental Section S4.3**. Additional quotations related to uncertainty about consequences and management of nitazene use.

1. “…Out of opioids and more don’t come for few days got couple 80mg oxycontin that I’ve split Into 2 to make them 40mg to hold off [withdrawal] and also pregablin and like 3 dirty 30s with [protonitazene] in. Can I take [buprenorphine] with [protonitazene] ?...”

**Supplemental Section S4.4**. Additional quotations about withdrawal symptoms, withdrawal management difficulties, and tolerance following nitazene use.

1. “i was using metonitazepyne. first day tired af, two days after stopping i thought i had covid or a [flu] or allergies because i was coughing and hot/cold/shivering, food not passing, and going to the toilets was abnormally easy. i also have clenched jaw … but i noticed weird but small pains in my tooths.”
2. “they really should because [suboxone] aren't going to [expletive] do anything for me”;
3. “Metonitazene can punch through Suboxone.”
4. “i have been using several different benzimidazole opioids including isotonitazene and etonitazene daily for approximately 9 - 10 months now and i've noticed that conventional opioids like heroin and fentanyl have little to no effect on my withdrawal symptoms anymore, no matter how high i dose them.”
5. “If you start using them regularly nothing else will touch you and tolerance rises so fast with them that you'll be spending much more than you ever did on heroin.”

**Supplemental Section S4.5**. Motivating factors for use and vascular injury associated with intravenous administration.

1. “…before i could nod off 30mg pharma oxy and now was only high with 60 but i think with 80+ i could still have caught a high. after a month of abuse i tried for two days feeling nothing to stop and then couldn't resist. i have mental issues and the relapse pattern always follow my mental health pattern, so i hope more stability and being more reliable might help on that and taking. i've changed from 0,06mg-0,09mg [metonitazepyne] intranasal, in 2 or mostly 3 times a day (4 weeks) to 0,03-0,06 if two times a day (more rare) boofed these last two weeks. i saw some improvement : diminution of moodiness, less tiredness, less cravings because longer highs. when i see how people tolerance skyrocketed i'm feeling lucky…”
2. “…realistically all of the zenes are not very [intravenously] safe. They all (except pyne and sometimes proto) needed a baking soda buffer to lessen the burn but even then they still burned out veins in days or weeks…”

**Supplemental Section S5**. Validation of the GPT-4o-based thematic forecasting pipeline on the held-out manually annotated sample.

To evaluate the reliability of the validated GPT-4o-based annotation pipeline used to infer full-dataset thematic frequencies, we compared automated predictions against the manually annotated sample after excluding two posts (post_id 12i4w99 and i5of4lj) that had been used during few-shot prompting. This yielded a held-out evaluation set of 291 manually annotated mentions. We evaluated each of the ten thematic labels using accuracy, precision, recall, F1 score, specificity, and balanced accuracy. We emphasize metrics beyond accuracy because several thematic labels are imbalanced, making accuracy alone potentially misleading. Bootstrap-based 95% confidence intervals for F1 are reported in **Supplemental Table S2**.

Across labels, average precision was 0.58, average recall was 0.65, average F1 was 0.57, average specificity was 0.87, and average balanced accuracy was 0.76. Label-specific performance varied. The highest F1 values were observed for preparation practices and route of administration (A; F1=0.72), potency and dose (C; F1=0.71), withdrawal and side effects experience (E; F1=0.66), uncertainty about nitazenes (H; F1=0.65), and source (B; F1=0.65). Lower F1 values were observed for general experience (D; F1=0.34), differences within nitazene class (F; F1=0.39), and use of other substances alongside nitazenes (I; F1=0.39).

Because of this label-specific variability, we interpret automated full-dataset outputs as predicted label frequencies rather than prevalence estimates and avoid strong inferences from lower-performing labels in the main text.

**Supplemental Section S6**. Uncertainty analysis of automated label performance.

To quantify uncertainty in automated label performance, we generated bootstrap-based 95% confidence intervals for the F1 score on the held-out manually annotated sample by repeatedly resampling the 291 evaluated mentions with replacement (samples of size 291 taken from the same set of 291 manually annotated mentions, but sampling with replacement enables these resamples to vary substantially) and recalculating F1 for each thematic label. These confidence intervals are reported in **Supplemental Table S2**. As expected, interval widths varied across labels and were generally wider for lower-support or lower-performing themes, including use of other substances alongside nitazenes (I; F1 95% CI: 0.226-0.528), international (J; 0.483-0.754), general experience (D; 0.231-0.442), and differences within nitazene class (F; 0.286-0.496), and narrower for higher-performing labels such as potency and dose (C; 0.641-0.783), source (B; 0.551-0.725), and uncertainty about nitazenes (H; 0.561-0.727). These findings reinforce that uncertainty in automated thematic classification is label-specific and support our decision to interpret full-dataset automated outputs descriptively as predicted label frequencies rather than prevalence estimates.

**Supplemental Tables**

**Supplemental Table S1**. Distribution of nitazene-related Reddit mentions across the most common subreddits represented in the extracted dataset.

| **Subreddit** | **Total mentions** | **Posts** | **Comments** | **General description / focus** |
| --- | --- | --- | --- | --- |
| Opioid_RCs | 10,136 | 1,095 | 9,041 | opioid / research chemical discussion |
| researchchemicals | 4,143 | 478 | 3,665 | research chemical discussion |
| opiates | 4,048 | 467 | 3,581 | opioid / opiate discussion |
| drogen | 1,752 | 177 | 1,575 | non-English general drug discussion |
| heroin | 1,301 | 81 | 1,220 | heroin / opioid discussion |
| Drugs | 835 | 218 | 617 | general drug discussion |
| fentanyl | 615 | 98 | 517 | fentanyl discussion |
| ObscureDrugs | 449 | 17 | 432 | discussion of uncommon / obscure psychoactive drugs |
| researchchemicals_DE | 448 | 100 | 348 | non-English research chemical discussion |
| OpiatesRecovery | 389 | 44 | 345 | opioid recovery discussion |
| canada | 369 | 2 | 367 | Canada-focused general discussion |
| Methadone | 306 | 21 | 285 | methadone treatment discussion |
| phenibut | 261 | 5 | 256 | phenibut discussion |
| drugsarebeautiful | 258 | 22 | 236 | drug-image / drug-identification discussion |
| suboxone | 215 | 26 | 189 | buprenorphine / Suboxone treatment discussion |
| australian | 205 | 3 | 202 | Australia-focused general discussion |
| Goblin | 190 | 9 | 181 | general drug discussion |
| brisbane | 179 | 4 | 175 | Brisbane-focused general discussion |
| benzodiazepines | 177 | 30 | 147 | benzodiazepine discussion |
| DrugNerds | 157 | 21 | 136 | drug pharmacology / information discussion |

**Supplemental Table S2**. Label-specific performance of the GPT-4o-based annotation pipeline on the held-out manually annotated sample (*N*=291 mentions).

| Theme name | Accuracy | Precision | Recall | F1 | Specificity | Balanced accuracy | Positive Rate | F1 95% CI |
| --- | --- | --- | --- | --- | --- | --- | --- | --- |
| Preparation practices and route of administration | 0.900 | 0.691 | 0.760 | 0.724 | 0.929 | 0.845 | 0.189 | 0.617-0.808 |
| Source | 0.811 | 0.758 | 0.562 | 0.645 | 0.921 | 0.741 | 0.227 | 0.551-0.725 |
| Potency and dose | 0.808 | 0.580 | 0.920 | 0.711 | 0.769 | 0.844 | 0.409 | 0.641-0.783 |
| General experience | 0.687 | 0.221 | 0.697 | 0.336 | 0.686 | 0.692 | 0.357 | 0.231-0.442 |
| Withdrawal and side effects experience | 0.893 | 0.789 | 0.566 | 0.659 | 0.966 | 0.766 | 0.131 | 0.535-0.772 |
| Differences within nitazene class | 0.725 | 0.255 | 0.867 | 0.394 | 0.709 | 0.788 | 0.351 | 0.286-0.496 |
| General comment | 0.808 | 0.627 | 0.521 | 0.569 | 0.900 | 0.711 | 0.203 | 0.470-0.667 |
| Uncertainty about nitazenes | 0.811 | 0.637 | 0.662 | 0.650 | 0.864 | 0.763 | 0.275 | 0.561-0.727 |
| Use of other substances alongside nitazenes | 0.880 | 0.647 | 0.275 | 0.386 | 0.976 | 0.626 | 0.058 | 0.226-0.528 |
| International | 0.918 | 0.571 | 0.690 | 0.625 | 0.943 | 0.816 | 0.120 | 0.483-0.754 |

***Note***: Balanced accuracy refers to the mean of recall and specificity, and positive rate refers to the proportion of held-out mentions predicted positive for the label.

**Supplemental Figures**

**Supplemental Figure S1**. Label-specific F1 scores of the GPT-4o-based annotation pipeline on the held-out manually annotated sample. Labels refer to the following thematic categories: A, Preparation practices and route of administration; B, Source; C, Potency and dose; D, General experience; E, Withdrawal and side effects experience; F, Differences within nitazene class; G, General comment; H, Uncertainty about nitazenes; I, Use of other substances alongside nitazenes; J, International.
